# The antioxidant activities, cytotoxic properties, and identification of water-soluble compounds of *Ficus deltoidea* leaves

**DOI:** 10.7717/peerj.5694

**Published:** 2018-10-11

**Authors:** Noor Nazirahanie Abrahim, Puteri Shafinaz Abdul-Rahman, Norhaniza Aminudin

**Affiliations:** 1 Department of Molecular Medicine, Faculty of Medicine, University of Malaya, Kuala Lumpur, Malaysia; 2 University of Malaya Centre for Proteomics Research (UMCPR), Faculty of Medicine, University of Malaya, Kuala Lumpur, Malaysia; 3 Institute of Biological Sciences, Faculty of Science, University of Malaya, Kuala Lumpur, Malaysia

**Keywords:** *Ficus deltoidea*, Antioxidant, Flavonoids, Phenolic acid

## Abstract

Leaves from three varieties of *Ficus deltoidea*, colloquially termed small- (FDS), medium- (FDM), and big-type leaf (FDB), were subjected to water extraction. The crude extracts were fractionated using water (WF) and ethyl acetate (EAF). The phenolic and flavonoid content, antioxidant activity, and cytotoxicity of the fractions were investigated. The EAF had the highest phenolic and flavonoid content compared to the other FDS fractions. Conversely, the FDM crude extract had the highest phenolic and flavonoid content compared to the other FDM samples. Antioxidant activity was highest in the FDB crude extract. Ultra-high–performance liquid chromatography showed that two compounds, vitexin and coumaric acid, were present in the FDB crude extract. Additionally, the *F. deltoidea* leaves caused no signs of toxicity in a normal liver cell line. Our findings show that *F. deltoidea* varieties have excellent antioxidant activity with no cytotoxic effects on normal liver cells.

## Introduction

It has been widely reported that various plant phytochemicals have biological activity and have the potential to affect disease risk through complementary mechanisms. A large number of these phytochemicals, particularly polyphenols, have been identified and have a wide range of biological activities, including neuroprotective effects (Zhu et al., 2007); inflammatory and immune response regulation (Rotelli et al., 2003); and anti-fungal (Raj et al., 2001), anti-cancer, hypoglycemic (Sharma, Chandrajeet & Partha, 2008), anti-hyperlipidemic (Ghule et al., 2006), and anti-atherosclerotic effects (Kim et al., 2005a). Despite such phytochemical activity, one of the main research interests is investigating their antioxidant potential for preventing or delaying autoxidation by free radicals. Free radical autoxidation leads to macromolecule deterioration, especially of lipids, proteins, carbohydrates, and DNA (Carocho & Ferreira, 2013). The oxidation of these macromolecules, particularly in humans, leads to various illnesses, such as Alzheimer’s disease, cancer, and cardiovascular diseases.

Due to its wide medicinal uses, we selected *Ficus deltoidea* for the present study. Also known as mistletoe fig or *Mas cotek*, *F. deltoidea* is a shrub that is native to Southeast Asia. It is a well-known shrub, especially among the Malays, and is used to treat diabetes, headache, sore throat, and cold. Its leaves have been studied for their hypoglycemic, antinociceptive (Sulaiman et al., 2008), anti-inflammatory (Abdullah et al., 2009), and antioxidant (Hakiman & Maziah, 2009) activity. However, its antioxidant activity has not been fully elucidated due to the presence of various bioactive compounds that might contribute to different antioxidant capacities. These complex mixtures, especially in plant extracts, can interact synergistically, additively, or antagonistically in different assays (Wang et al., 2011; Colon & Nerin, 2016). According to Misbah, Abdul Aziz & Aminudin (2013), a combination of assays incorporating various mechanisms of action would be very helpful for providing complete information on the antioxidant capacity of a specific plant. Thus, the aim of the present study is to determine the antioxidant capacity of *F. deltoidea* leaves in different *in vitro* systems as well as to determine their cytotoxic effect on a normal liver cell line.

## Methods

### Sample preparation

The leaves of three varieties of *F. deltoidea* were obtained from a plantation in Rembau, Negeri Sembilan. The *F. deltoidea* varieties (small, FDS; medium, FDM; big, FDB) were deposited in the Herbarium, Rimba Ilmu, University of Malaya, Kuala Lumpur, and assigned individual voucher specimen numbers (KLU046467, KLU046469, KLU046471, respectively). The leaves were rinsed and air-dried at room temperature until they reached a constant weight and then ground into powder using a commercial blender. The powder was kept at −20 °C for further analysis.

### Extraction and liquid–liquid fractionation

The dried leaf powder underwent extraction according to Misbah, Abdul Aziz & Aminudin (2013) to yield the crude extract. Then, the crude extracts were fractionated using partial liquid–liquid separation for finer separation of the plant constituents into fractions of different polarity. The process involved the use of two immiscible solvents of different polarities, i.e., water and ethyl acetate using the method established by Misbah, Abdul Aziz & Aminudin (2013) to yield the water and ethyl acetate fractions. Subsequent experiments were conducted using the FDS, FDM, and FDB crude extracts along with their respective water and ethyl acetate fractions.

### Ultraviolet–visible (UV-Vis) spectroscopy

UV-Vis spectroscopy was used to distinguish the presence of phenolic components in the samples. The UV-Vis absorption pattern of phytoconstituents can be measured in very dilute solution against a solvent blank using a UV-Vis spectrophotometer. Sample solutions prepared in water were used for this analysis, and the spectra were recorded against a control (water). The wavelength maxima (*λ*_max_) of each samples was recorded.

### Extract analysis assays

#### Folin-Ciocalteu assay

The total polyphenolic content (TPC) of the samples was determined using the Folin-Ciocalteu assay. Folin-Ciocalteu reagent (0.1 ml) was added to 1 µl sample and incubated for 5 min. Sodium carbonate (0.07 ml) was added to the mixture and left in the dark for 2 h. The absorbance of the mixture was measured at 765 nm using a microplate reader (BioTek, USA). Gallic acid (0–200 µg/ml) was used as the standard and was processed under similar conditions as above. The TPC in the samples was expressed as mg gallic acid equivalents (GAE)/g dry weight. All experiments were carried out in triplicate.

#### Aluminum chloride assay

Quercetin (0–100 µg/ml) was prepared to generate the standard curve. Sample (500 µl) or quercetin (1 mg/ml) was combined with 95% ethanol (1.5 ml), 10% aluminum chloride (0.1 ml), 1 M potassium acetate (0.1 ml), and distilled water (2.8 ml). The absorbance of the mixture was determined at 415 nm after 30-min incubation. The total flavonoid content (TFC) was expressed as mg quercetin equivalents (QE)/g dry weight. All analyses were performed in triplicate.

#### Cupric ion (Cu^2+^) reducing antioxidant capacity (CUPRAC) assay

The CUPRAC assay is the most commonly used assay for *in vitro* determination of the antioxidant activity of food elements, biological fluids, and also plant extracts. It uses copper (II)-neocuproine [Cu(II)-Nc] as the oxidizing agent to measure antioxidant activity close to physiological pH conditions. For this assay, 10 mM copper solution (1 ml) was mixed with 7.5 mM neocuproine (1 ml), 1 M ammonium acetate buffer (1 ml), and sample (1 ml), incubated for 30 min, and the absorbance of the mixture was determined at 450 nm. The samples and the positive control quercetin (0–1,000 µg/ml) were tested. All experiments were performed in triplicate.

#### 2,2-Diphenyl-1-picryl-hydrazyl (DPPH) assay

We determined the radical scavenging activity of antioxidants in the samples and quercetin (positive control, 0–1,000 µg/ml) using DPPH. Samples (100 µl) were added to 600 µl DPPH reagent and mixed vigorously. The mixture was incubated in the dark for 30 min at room temperature, following which the decrease in absorbance was detected at 517 nm. The same procedure was repeated with the positive control. The absorbance of the radical without antioxidants was used as the negative control. The experiment was carried out in triplicate, and the percentage of inhibition (%) was calculated using the following formula: [absorbance_(blank)_ –absorbance_(sample)_/absorbance_(blank)_] ×100.

#### Non-enzymatic lipid peroxidation (thiobarbituric acid–reactive substances, TBARS) assay

Fowl egg yolk homogenate was used as the lipid-rich medium and underwent non-enzymatic peroxidation when incubated with ferrous sulphate (FeSO_4_), which act**s** as a mediator for the initiation of lipid peroxidation. The yolk was separated from the albumin and the yolk membrane was removed. We used 0, 0.1, 0.5, 1, and 2 mg/ml sample and positive control were used. Samples (100 µl) were mixed with 500 µl buffered egg yolk (1%) and 100 µl FeSO_4_ (1 M) in a test tube. The mixture was incubated at 37 °C for 1 h, and then 250 µl trichloroacetic acid (TCA,15%) and 500 µl TBA (1%) were added to the mixture. Subsequently, the mixture was heated for 10 min at 100 °C and left to cool, centrifuged at 3,500 rpm for 10 min, and its absorbance was detected at 532 nm. Each experiment was carried out independently in triplicate. The percentage of inhibition (%) was calculated using the following formula: [absorbance_(blank)_—absorbance_(sample)_/absorbance_(blank)_] ×100.

#### Ferrous ion chelating (FIC, ferrozine) activity assay

The FIC activity assay was used to investigate the FIC capacity of the samples (Singh & Rajini, 2004). Briefly, 2 mM FeSO_4_ (0.005 ml) was mixed with 0–400 µg/ml sample (0.1 ml), followed by the addition of 5 mM ferrozine (0.02 ml). The absorbance of the reaction mixture was detected at 562 nm after 10-min incubation. A higher absorbance at 562 nm indicated the weaker FIC strength of the chelator. Ethylenediaminetetraacetic acid disodium salt (EDTA-Na_2_) was used as the reference standard (Diaz et al., 2012). A blank, containing water, was also incorporated under the same conditions. The FIC capacity of the extracts (%) was estimated using the following equation: [(absorbance_[blank]_ –absorbance_[test])_/absorbance_(blank)_] ×100, where absorbance_test_ is the absorbance of the reaction mixture containing the extract or ascorbic acid and absorbance_blank_ is the absorbance of the blank. All determinations were carried out in triplicate.

#### Ferricyanide (Prussian blue) assay

The reducing power of the crude extracts and fractions were measured using the method of Bursal & Koksal (2011) with a slight adjustment. The formation of the Prussian blue complex is due to the reduction of ferric ions (Fe^3+^) to ferrous ions (Fe^2+^), the absorbance of which can be detected at 700 nm. Sample (0, 0.1, 0.5, 1 mg/ml) was mixed with sodium phosphate buffer (0.2 M, pH 6.6) and potassium ferricyanide (1%). The mixture was incubated at 50 °C for 20 min using a dry bath, followed by the addition of TCA (10%) and ferric trichloride (FeCl_3_, 0.1%) to the reaction mixture. Distilled water was used as the blank. The absorbance of the mixture was detected at 700 nm using a UV spectrophotometer.

### Toxicity study

The inhibition of cell growth was measured using the 3-(4,5-dimethylthiazol-2-yl)-2,5-diphenyltetrazolium bromide (MTT) assay. MTT is a tetrazolium salt that is cleaved into formazan crystals by succinate dehydrogenase and is only active in viable cells. A higher amount of formazan dye produced indicates a higher number of viable cells. WRL68 normal liver cells were seeded in 96-well culture plates (5 × 10^3^ cells/well) and left to attach overnight at 37 °C in a 5% CO_2_ atmosphere. *F. deltoidea* leaf crude extracts and fractions were added to each well to yield final concentrations of 50, 100, 200, and 500 µg/ml. The cells were also treated with the same amount of vehicle (water) present in the plant extracts, and this was used as the negative control. Cytotoxicity was measured after 24-, 48-, and 72-h treatment by adding 10 µl MTT reagent (5 mg/ml) to the cells and incubating them for an additional 4 h. Finally, the medium and MTT reagent were discarded and replaced with 100 µl isopropanol. The absorbance was detected at 595 nm and the percentage of inhibition (%) was calculated as follows: [(total cells –  viable cells)/total cells] ×100.

The median inhibitory concentrations (IC_50_) were determined. All experiments were carried out in three separate batches, each in triplicate.

### Phytochemical analysis and identification

The sample with the most activity was subjected to ultra-high–performance liquid chromatography (UHPLC) identification. The sample was prepared using two hydrolysis methods: acidic and alkaline. The acidic hydrolysis was prepared by mixing 10 mg sample with 1.2 M hydrochloric acid (HCl) in 50% methanol, while alkaline hydrolysis was performed by mixing 10 mg sample with 0.5 M sodium hydroxide (NaOH) in 50% methanol. Both mixtures were heated for 2 h at 90 °C using a dry water bath (Labnet, USA), left to cool, and centrifuged at 5000 rpm for 20 min. The supernatant was filtered and stored at −20 °C until used.

The hydrolyzed samples were analyzed using a UHPLC system (Agilent, Santa Clara, CA, USA) comprising a dual wavelength absorbance detector, quaternary pumps, auto-injector with a 6-µl sample loop, and a column oven. Reverse-phase separations were carried out at 30 °C using a ZORBAX C18 column (Agilent, Santa Clara, CA, USA) (3. 9 × 50 mm). Trifluoroacetic acid (TFA) in water at pH 2.6 (solvent A) and acetonitrile (solvent B) were used as the mobile phase. The flow rate was maintained at 0.3 ml/min for a total run of 9 min, and the gradient program consisted of 5% to 15% solvent B for 3 min, 15% to 50% solvent B for 3 min, and 50% to 100% solvent B for 2 min, and it then was reduced to initial conditions for another 1 min. The eluted peaks were detected at 280 nm and 335 nm. The samples were diluted in solvent A to yield 5% methanol, and 3 µl sample was injected into the UHPLC system. All samples were prepared and analyzed in triplicate. The standards used in the UHPLC analysis were coumaric acid, catechin, gallic acid, and vitexin. Standard stock solutions of each compound were prepared in methanol at 1 mg/ml. A mixture of the standard solutions was prepared at various concentrations (100–500 ng, and 3 µl was injected into the UHPLC and run using the conditions described above. Peak identification was carried out by spiking the samples with the standards of possible compounds and by spectral analysis. The UV spectra of individual peaks were recorded in the 200–400-nm range. Data acquisition and processing were performed using a Lab Advisor chromatography manager (Agilent, Santa Clara, CA, USA).

### Statistical analysis

The data were analyzed using the Excel statistical package for Windows software (Microsoft, USA); all analyses were done in triplicate. The results are expressed as the mean ± standard deviation (SD). The correlation coefficient was used to detect the relationship between the extracts’ phenolic content and the antioxidant activity. Statistical significance was calculated using Student’s *t*-test. Differences between means at the 95% confidence level (*p* < 0.05) were considered statistically significant as compared to the control.

## Results

### UV-Vis spectrophotometer analysis and polyphenol and flavonoid content in *F. deltoidea* leaf extracts and fractions

Table 1 shows the yield of extractible components and polyphenolic and flavonoid contents of the *F. deltoidea* leaf crude extracts and fractions. The yield of extractible components, expressed as g/100 g dried weight, ranged from 17 g/100 g dried weight (FDB crude extract) to 0.41 g/100 g dried weight (FDM ethyl acetate fraction). The TPC detected in the samples ranged 10–55 mg GAE/g dry weight. Among the crude extracts, the FDM crude extract had the highest phenolic content, followed by that of FDB and FDS. Fractionation of crude extracts causes changes in the phenolic content pattern. The FDS ethyl acetate fraction had the highest TPC (Table 1), followed by that of the FDS water fraction and FDS crude extract. FDM and FDB exhibited a different pattern whereby the crude extracts from both varieties had the highest TPC, followed by that of the water and ethyl acetate fractions. Contents in the water and ethyl acetate fractions, however, were not significantly different. The TFC was highest in the FDB ethyl acetate fraction compared to that of FDM and FDS. The findings show that, in all varieties tested, the ethyl acetate fraction had a higher concentration of flavonoids in comparison to the water fraction.

**Table 1:** Yield of extractible components, polyphenolic and flavonoid contents of the extract and fractions of different varieties of *Ficus deltoidea* leaves.

Sample	Extract/fraction	Yield of extractible components (g/100 g of dried weight)	Phenolic content* (mg GAE/g of dry weight)	Flavonoid content* (mg QE/g of dry weight)
FDS	Crude	14.71	9.75 ± 0.70	0.65 ± 0.00
	Water fraction	3.48	11.20 ± 1.74	5.05 ± 0.00
	Ethyl acetate fraction	0.59	54.46 ± 0.76	165.05 ± 0.01
FDM	Crude	11.79	43.23 ± 0.45	163.47 ± 0.01
	Water fraction	2.84	19.61 ± 0.87	61.37 ± 0.00
	Ethyl acetate fraction	0.41	16.42 ± 0.55	148.74 ± 0.00
FDB	Crude	17	39.02 ± 1.95	101.37 ± 0.00
	Water fraction	3.95	21.13 ± 0.43	59.79 ± 0.00
	Ethyl acetate fraction	0.68	18.09 ± 0.38	212.42 ± 0.00

**Notes.**

Results were expressed as means ± S.D. (*n* = 3).

GAE gallic acid equivalent QE quercetin equivalent

**Figure 1:**
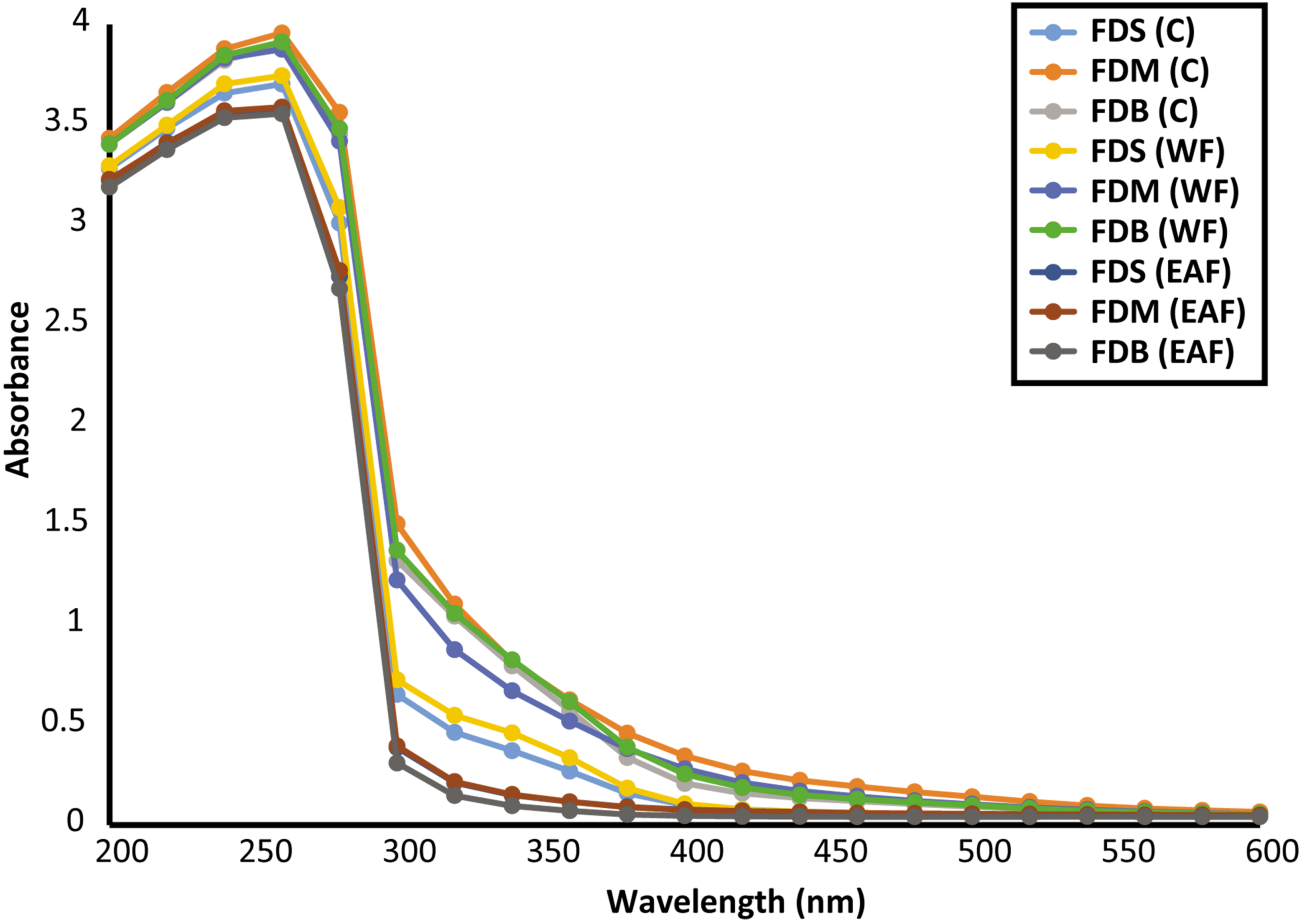
UV-vis spectra of crude and fractions of *F. deltoidea* leaves.

For further characterization of the phenolic compounds, the samples’ UV–Vis absorption spectra were assessed at 200–600 nm. Figure 1 shows that all samples had *λ*_max_ of 250–300 nm, which may have been due to the presence of flavone/flavonol derivatives or anthocyanins, with absorbance values peaking at 4.

### *F. deltoidea* leaf crude extract and fractions antioxidant activity

**Figure 2:**
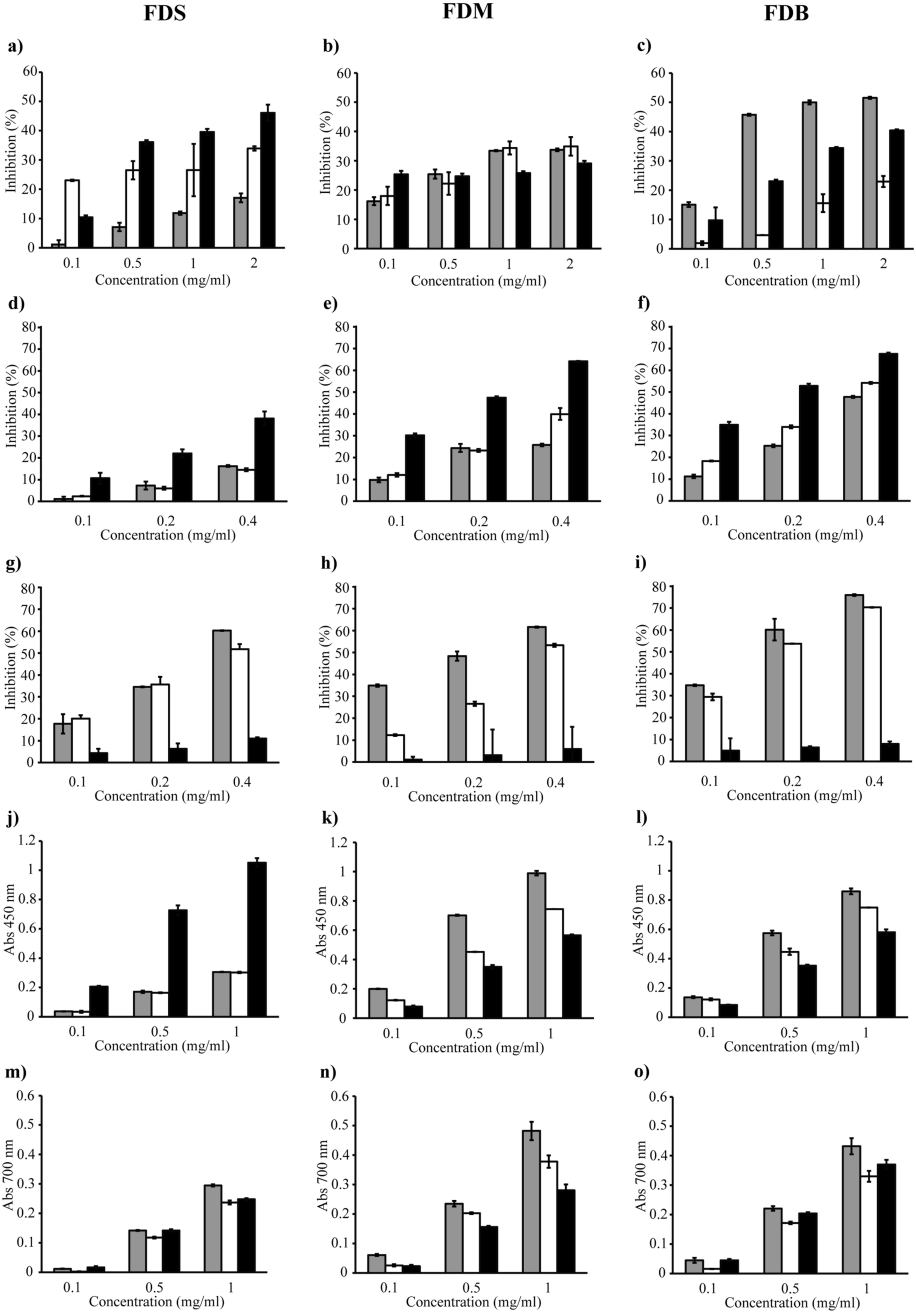
FDS, FDM and FDB were tested for various antioxidant activities which are lipid peroxidation (A–C), DPPH radical scavenging (D–F), ferrozine (G–I), CUPRAC (J–L) and ferricyanide (M–O) assays. Crude extract (grey bar) Water fraction (white bar) Ethyl acetate fraction (black bar).

In this study, spectrophotometric TBA assay was used to evaluate the ability of the crude extracts and fractions to inhibit lipid peroxidation (Figs. 2A to 2C). Figure 2C demonstrates that, unlike the other samples, only the FDB crude extract caused at least 50% inhibition of lipid peroxidation.

In the DPPH assay, the discoloration of the reaction mixture reflects the potency of the antioxidant in the tested sample. Figures 2E and 2F shows that only FDB and FDM samples showed good inhibition of DPPH radicals, as both varieties inhibited at least 50% of the radicals. Fractionation of the crude extract helped improve the scavenging activity, as the FDB and FDM ethyl acetate fractions showed potent activity, with lower IC_50_ of 182 µg/ml and 223 µg/ml, respectively. This indicates that the compounds with strongest radical-scavenging ability in FDB and FDM are of medium polarity. However, both samples were not as effective as the positive control, quercetin. FDS crude extract and its fractions (Fig. 2D) had the weakest DPPH radical–scavenging activity, indicating the lack of hydrogen-donating ability. Overall, the samples’ DPPH radical–scavenging activity was in the order of FDB >  FDM >  FDS.

The metal chelating ability of the samples were tested using the ferrozine assay, where lower absorbance indicates stronger FIC strength of the tested sample. Figures 2G to 2I shows that all crude extracts exhibited good FIC activity. In fact, the FDB crude extract (Fig. 2I) exhibited the highest FIC activity by far, with a chelating IC_50_ of 150 µg/ml. Generally, the Fe^2+^ chelating activity of the crude extracts was in the order of FDB >  FDM >  FDS. All water fractions also showed FIC activity; however, the IC_50_ values were rather high compared to their respective crude extracts. In addition, all ethyl acetate fractions exhibited very low FIC activity, with no IC_50_. Even though the extracts did not chelate Fe^2+^ as strongly as EDTA, they demonstrated noteworthy chelating properties.

In the CUPRAC assay, a higher absorbance indicates a higher Cu^2+^-reducing power. Figures 2J to 2L shows that all samples reacted with the reagent. The FDS ethyl acetate fraction exhibited the most Cu^2+^-reducing activity, while the FDS crude extract and water fraction had the lowest activity (Fig. 2J). A similar pattern of activity was observed for FDM and FDB (Figs. 2K and 2L) in the order of crude extract > water fraction > ethyl acetate fraction. The reducing activity of both the FDM and FDB crude extracts might have been due to the presence of various phytochemicals that interacted synergistically. On the other hand, fractionation causes the loss of reducing activity, which could be observed in both the FDM and FDB fractions.

The ferricyanide assay is a reducing power assay based on the ability of test samples to reduce yellow Fe^3+^ to blue Fe^2+^. The resulting blue color is considered linearly connected to the total reducing capacity of electron-donating antioxidants. Figures 2M to 2O shows that the reducing activity could be divided into three types: high, moderate, and low. The extracts that exhibited the highest reducing activity were the FDM crude extract (0.482), followed by the FDB crude extract (0.432). The samples that showed moderate activity were the FDM water fraction (0.378), FDB ethyl acetate fraction (0.370), and FDB water fraction (0.330). The samples that exhibited low reducing abilities were in the order of FDS crude extract >  FDM ethyl acetate fraction > FDS ethyl acetate fraction >  FDS water fraction.

### Correlation coefficient between antioxidant assays

Table 2 shows the relationship between the TPC and TFC and the antioxidant activities of each sample. Strong interaction was observed between the TPC and Cu^2+^-reducing activity (*R*^2^ = 0.9161, *p* < 0.01) and between the TFC and DPPH radical–scavenging activity (*R*^2^ = 0.7765, *p* < 0.05). There were moderate correlations between the TFC and Cu^2+^-reducing activity (*R*^2^ = 0.5867, *p* < 0.01) and between the TFC and lipid peroxidation activity (*R*^2^ = 0.5225, *p* < 0.05). In contrast, the TPC was poorly correlated with DPPH radical–scavenging and metal-chelating activity (*R*^2^ = 0.1697, *p* < 0.05; *R*^2^ =  − 0.0213).

**Table 2:** Correlation analyses between phenolic (PC) and flavonoid content (FC) and antioxidant activities of the crude extracts and fractions of *F deltoidea* leaves.

	DPPH assay	Cuprac assay	Ferrozine assay	Ferricyanide assay	Lipid peroxidation assay
	*R*^2^	*R*^2^	*R*^2^	*R*^2^	*R*^2^
PC	0.1697^*^	0.9161^**^	−0.0213	0.3255^**^	0.6658
FC	0.7765^*^	0.5867^**^	−0.6321^*^	0.3358^**^	0.5225^*^

**Notes.**

* Data with *p* value < 0.05 were considered significant.

** Data with *p* value <  0.01 were considered significant.

### Effects of *F.  deltoidea* leaf extracts on WRL68 cell growth

Normal liver cell, WRL68 was used for toxicity evaluation. Table 3 summarizes the results of the cytotoxic activity of the *F. deltoidea* leaf extracts. The data are expressed as the IC_50_ for all incubation times. No IC_50_ was detected for FDS and FDM even up to 72-h incubation. On the other hand, the FDB crude extract inhibited 50% of the cells after 72-h incubation, with an IC_50_ of 340 µg/ml. The WRL68 cells were also more sensitive to the FDB water fraction, which had IC_50_ of 375 µg/ml, 300 µg/ml, and 227 µg/ml after 24-, 48-, and 72-h incubation, respectively.

**Table 3:** Cytotoxicity of the extracts of *F. deltoidea* leaves against WRL68 cells.

Sample	Extract/fraction	Incubation		
		**24 hr** (IC_**50**_ - µg/ml)	**48 hr** (IC_**50**_ - µg/ml)	**72 hr** (IC_**50**_ - µg/ml)
FDS	Crude	N/D	N/D	N/D
	Water fraction	N/D	N/D	N/D
	Ethyl acetate fraction	N/D	N/D	N/D
FDM	Crude	N/D	N/D	N/D
	Water fraction	N/D	N/D	N/D
	Ethyl acetate fraction	N/D	N/D	N/D
FDB	Crude	N/D	N/D	347.67 ± 2.52
	Water fraction	378.3 ± 5.51	306.7 ± 7.37	224 ± 21.17
	Ethyl acetate fraction	N/D	N/D	N/D

**Notes.**

The experiment was conducted in a 96-well plate, each in triplicate. Cells were allowed to attach for 24 h after seeding. WRL68 cells were treated with various concentrations of the extracts of *F.deltoidea* crude extracts and fractions for 24, 48 and 72 h.

Results were expressed as means ± S.D. (*n* = 3).

IC_50_ = concentration of plant extracts (μ g/ml) that inhibited 50% of the cells.

N/D, no inhibition detected

**Figure 3:**
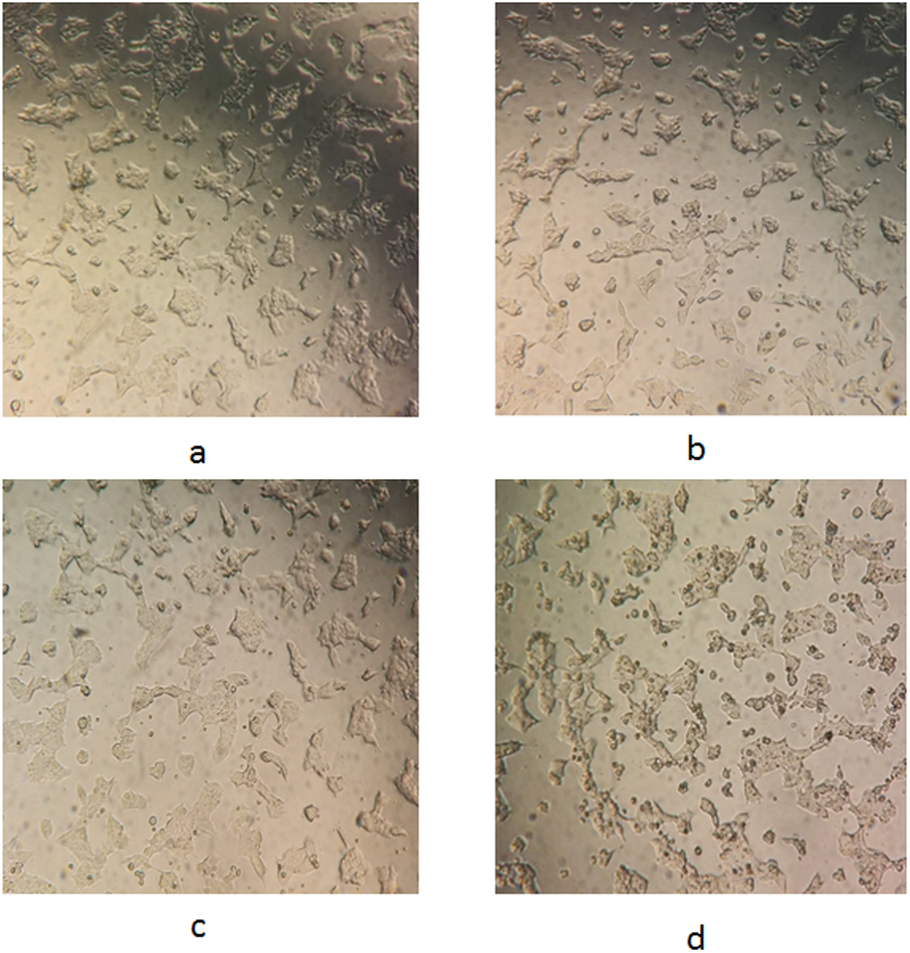
Representative images for morphological changes of WRL68 cells after 72 h of incubation without (A) and with treatment of FDS (B), FDM (C) and FDB (D) at the highest concentration (original magnification:10×).

The morphological changes of the WRL68 cells following treatment with the *F. deltoidea* leaf extracts were observed using a phase contrast microscope after 72-h incubation. Figure 3 shows that there was an obvious difference between the untreated (Fig. 3A) and treated (Figs. 3B–3D) cells. The distinct changes observed in the cells treated with the FDB crude extract (Fig. 3D) included shrinkage, rounding, and detachment from the surface of the wells. These alterations became increasingly noticeable as the dose increased, but were not observed in the control cells. In contrast, the FDS and FDM extracts (Figs. 3B and 3C) showed no indications of cytotoxicity, as no morphological changes were observed.

### UHPLC phytochemical analysis of *F. deltoidea* leaves

**Figure 4:**
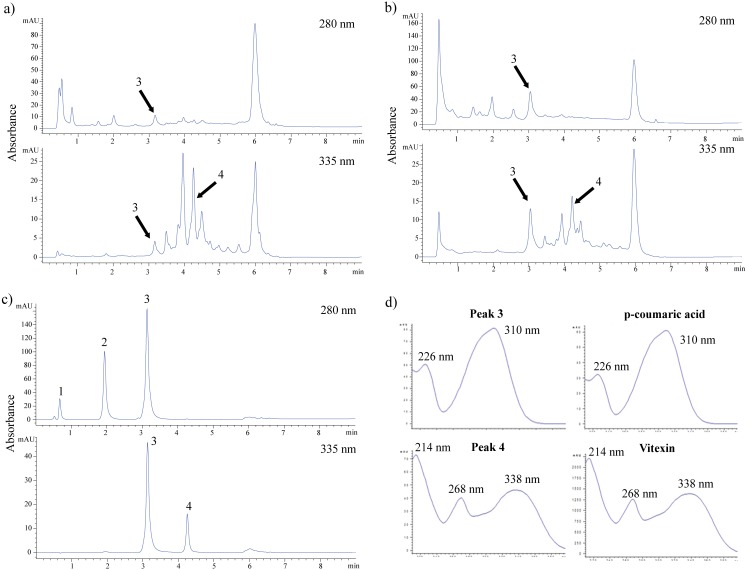
HPLC chromatograms of crude water extract of FDB in acid (A), alkaline (B) hydrolysis and standard solution mixture (C). (D) UV-spectra of peak 3 and 4 corresponding to *p*-coumaric acid and vitexin. These compounds were detected at a total run of 9 min by using two different wavelength at 280 and 335 nm. Standard solution mixture consists of gallic acid (1), catechin (2), *p*-coumaric acid (3) and vitexin (4).

The sample with good activity in most antioxidant assays was subsequently subjected to UHPLC for phytochemical identification. Figure 4C depicts the separation of a standard mixture of four flavonoids and phenolic acids. Good dissolution was obtained in a short separation time of 9 min. Gallic acid, catechin, and *p*-coumaric acid were detected at 280 nm. The absorbance at 335 nm was used to detect the presence of coumaric acid and vitexin.

Figure 4A and 4B show the chromatogram of the FDB crude extract under acidic and alkaline hydrolysis conditions, respectively. Two peaks were positively identified based on their retention time, UV spectra, and commercial standards spiking test. Coumaric acid was present in a higher concentration under alkaline conditions as compared to acidic conditions. In contrast, there was a higher amount of vitexin following acidic hydrolysis rather than alkaline hydrolysis. Figure 4D shows that the UV spectrum of *p*-coumaric acid was characterized by the presence of two maxima at 226 nm and 310 nm, while the vitexin spectrum consisted of a prominent band at 214 nm and 268 nm, with a shoulder at the 338 nm region.

## Discussion

In the present study, water was used as a medium for extracting the hydrophilic antioxidants present in *F. deltoidea* leaves. This is of interest, as typically in the preparation of food and nutraceuticals, aqueous plant extracts are nutritionally more useful and have apparent benefit in relation to safety. On top of that, this is also a similar method as how the plant extract was prepared and consumed traditionally. Many researchers have found that the physiological functions of natural foods can be associated with the presence of phenolic components. Furthermore, flavonoids have various biological properties, such as anti-bacterial, anti-inflammatory, anti-viral, and anti-thrombotic effects. Therefore, it is reasonable to determine the TPC and TFC contents of the *F. deltoidea* leaf extracts. Three most commonly found varieties of *F. deltoidea* were evaluated in this study and the results show that some of our findings differ from that of others. Pushpanathan & Nithyanandam (2015) reported that the *F. deltoidea* leaf TPC was 96.225 mg GAE/100 g dried weight (water extract), 225.917 mg GAE/100 g dried weight (80% methanol), and 264.765 mg/100 g dried weight (80% ethanol), which was lower than that detected in our sample. By contrast, Mun et al. (2017) detected higher *F. deltoidea* leaf TPC compared that in the present study, i.e., 368.42 ±  6.37 mg GAE/g (aqueous extract), 295.03 ± 16.65 mg GAE/g (methanol extract), and 263.45 ±  5.28 mg/g (ethanol extract). By contrast, we detected higher TFC as compared to others (Hakiman & Maziah, 2009; Dzolin et al., 2010; Soib et al., 2015). Thus, it is difficult to compare the results obtained from different investigations, as differences may arise for various reasons. Differing extraction methods are a factor in the varied TPC and TFC determinations between studies. Various methods can be used to extract plant compounds. Parameters such as the nature and volume of the solvent, temperature, and time can affect compound extraction (Soong & Barlow, 2004; Maisuthisakul & Pongsawatmanit, 2004). Moreover, the presence of interfering substances in plant extracts, such as lipophilic compounds, sugar, ascorbic acid, and aromatic amines may contribute to the variations in TPC estimation between studies (Ikram et al., 2009; Khan, Bakht & Shafi, 2016). It is also important to note that the TPC and TFC evaluation methods are based on the general structure of phenolics and flavonoids; and hence, complexity of the compounds and structural modifications that could have occurred during growth process may lead to variations. The choice of using different varieties also will results in differences in term of compound extracted, their types and quantity. Each of this *Ficus* variety differs in term of morphology and growth behavior and requirements, all of which that may contribute to compound variations. Eight varieties of *F. deltoidea* can be found in Malaysia; thus, genetic and geographical origins may also affect the chemical composition between plants (Zimisuhara et al., 2015; Chen, Wang & Chen, 2014).

Many methods can be used to demonstrate the antioxidant activity of plant extracts. However, no single assay can establish the complete antioxidant potential of such compounds, as multiple reactions and mechanisms are involved in the antioxidative processes (González-Centeno et al., 2013). Hence, we tested the antioxidant activity of *F. deltoidea* leaves using several assays involving different mechanisms. Fractionation of the crude extracts led to the loss of antioxidant activity, especially for FDM and FDB. The finding suggests that the bioactive components in the crude extracts may act synergistically to produce the antioxidant effects, and fractionation might have eliminated some of the compounds (Zahin, Aqil & Ahmad, 2010). In fact, the crude extracts’ TPC and TFC (Table 1) correlated well with the antioxidant activity (Fig. 2), but not that of the fractions. Furthermore, the potential antioxidants in both FDM and FDB were mainly high-polarity compounds. Antioxidant activity was also observed for the FDS ethyl acetate fraction, which was also due to the presence of high TPC and TFC. This suggests that medium-polarity compounds contribute more to that particular activity. Based on these observations, we could see differences in antioxidants capability showed by the varieties evaluated; FDB and FDM demonstrated a more similar pattern contrasting with FDS. This may also suggests that antioxidant effects of *F.deltoidea* could be contributed by variety of compounds other than the commonly known phenolics. Nevertheless, Table 2 shows the lack of correlation between the TPC and TFC and the antioxidant activity. Thus, highlighting that the phenolic and flavonoid compounds were not major contributors to the antioxidant activity of *F. deltoidea* leaves. Our findings are in agreement with the studies of Norra (2011) and Mun et al. (2017). The differing antioxidant activity of the samples was most probably due to the differing phenolic content and phenolic composition. In some instances, samples with lower phenolic and flavonoid content had higher antioxidant activity. In plant extracts, the types and amounts of phenolics and flavonoids present does not necessarily affect the antioxidant activity; in fact, it also depends on the degree of polymerization, concentration, and the synergistic interaction between the diverse chemical structures of the antioxidants and the antioxidant assays (Stratil, Klejdus & Kubaan, 2006; Sulaiman et al., 2011). Furthermore, antioxidants can exert their protective effects at different stages of oxidation and through different mechanisms (Parejo et al., 2002; Wang et al., 2011; Chanudom et al., 2014).

Despite the good antioxidant activity in different assay systems, determining the extracts’ toxicity, especially in a normal cell line, was essential. Here, we tested the cytotoxicity of the *F. deltoidea* leaf crude extracts and fractions on the WRL68 normal liver cell line. The liver is the main site of various metabolism activities in the human body, thus it is of interest to test the extracts’ cytotoxicity using this cell line. To the best of our knowledge, ours is the first study to analyze the cytotoxicity of *F. deltoidea* leaves in a normal liver cell line. As both FDM and FDS showed no signs of cytotoxicity (Table 3), this could be interpreted as a positive sign in that they are relatively non-toxic, rendering them safe for consumption as an alternative medicine and health supplement. However, the US National Cancer Institute (NCI) states that a plant extract is considered toxic if it causes cytotoxicity with IC_50_ <  20 µg/ml (Shafaei et al., 2014). Thus, FDB also can be considered non-toxic to WRL68 cells. The samples were also found to be non-toxic on MRC5 (human lung fibroblast) and Chang (liver) cell lines (Data S2). Another study also reported that *F. deltoidea* leaves are not toxic to normal cell lines, particularly human umbilical vein endothelial cells (HUVEC) and neuroblastoma cells (SH-SY5Y) (Dzolin et al., 2010; Shafaei et al., 2014). These data are also concurrent with Ilyanie, Wong & Choo (2011), who reported that *F. deltoidea* leaf extract does not induce liver and kidney toxicity in streptozotocin (STZ)-induced diabetic rats. However, *F. deltoidea* leaves are toxic to other cell lines, especially human cancer cell lines, such as HL-60 (leukemia), DU145 (prostate cancer), HCT116 (colorectal carcinoma), and MDA-MB-231 (hormone-resistant breast cancer) (Soib et al., 2015; Shafaei et al., 2014; Norrizah et al., 2012). In contrast, Lee et al. (2011) found that *F. deltoidea* leaf did not cause toxicity in MCF-7 breast cancer cells. These reports show that *F. deltoidea* leaves have different toxicity effects on different cell types.

Based on the consistent antioxidant activity shown in each antioxidant assay and because it was non-toxic to normal cells, the FDB crude extract was subjected to UHPLC identification. Acid- and alkaline-catalyzed hydrolysis were used to release flavonoids and phenolic acids from their bound forms for easier identification. Coumaric acid and vitexin were detected using a method developed in our laboratory. The presence of vitexin in the *F. deltoidea* leaves was in accordance with that reported previously (Abdullah et al., 2009; Omar, Mullen & Crozier, 2011; Choo et al., 2012). In the present study, vitexin (peak 4) was easily detected in acidic medium at 335 nm (Fig. 4A). Moreover, several peaks could not be identified at retention times of 3.43 min, 3.92 min, and 4.43 min. These peaks showed UV maxima at 335 nm and 270 nm, suggesting that these compounds are flavonoids with apigenin derivatives. On the other hand, vitexin and other peaks showed sign of degradation in alkaline medium (Fig. 4B), as lower amounts of vitexin were detected. Figure 4B shows that alkaline hydrolysis was a good medium for liberating *p*-coumaric acid (peak 3), as *p*-coumaric acid is degraded in acidic conditions (Verma, Hucl & Chibbar, 2009; Waszkowiak & Gliszczyńska-Świgło, 2016). This may explain the lower amount of *p*-coumaric acid we detected following acid hydrolysis. Previous work on the phytochemical composition of *F. deltoidea* leaves has also reported the presence of other compounds such as ursolic acid, epicatechin, naringenin, catechin, epigallocatechin, luteolin, and coumaroylquinic acid (Shafaei et al., 2014; Omar, Mullen & Crozier, 2011). Several researchers have also found vitexin in other plant species such as *Acer palmatum*, *Vitex agnus castus*, *Trigonella foenum-graecum* L., *Arum dioscoridis*, and *Codiaeum variegatum*. This flavone is a compound that contributes to the antioxidant activity of these plants, particularly for the DPPH radical scavenging, lipid peroxidation, and ferric reducing assays (Kim et al., 2005b; Gokbulut et al., 2010; Uguzlar, Maltas & Yildiz, 2012; Khole et al., 2014; Hassan et al., 2014). *p*-Coumaric acid is a phenolic acid that prevents lipid peroxidation and scavenges DPPH radicals (Kiliç & Yeşilouğlou, 2013; Shairibha & Rajadurai, 2014; Widowati et al., 2016). Thus, it is suggested that the FDB crude extract activity could have been due to the presence of vitexin and *p*-coumaric acid.

There have been very few studies on *F. deltoidea* compounds and their effects on biological activity. Lupeol in *F. deltoidea* leaves exhibited antibacterial activity when tested on three bacteria: *Escherichia coli*, *Bacillus subtilis*, and *Staphylococcus aureus* (Suryati et al., 2011). Vitexin and isovitexin have also been detected in *F. deltoidea* leaves and have antidiabetic properties by inhibiting *α*-glucosidase activity in STZ-induced diabetic rats (Choo et al., 2012). Hanafi et al. (2017) found that a mixture of compounds comprising oleanolic acid, botulin, and lupeol in the active fraction of *F. deltoidea* showed better anti-proliferative activity compared to individual compounds. The active fraction exerted its anti-proliferative properties by increasing the expression of Bax and Smac/DIABLO (diablo IAP-binding mitochondrial protein) and downregulating the expression of Bcl-2 in PC3 prostate cancer cells, which leads to apoptosis.

## Conclusion

Through *in vitro* assays involving different mechanisms, such as radical scavenging, metal chelation, reduction, and suppression of the initiation of radical formation, the present study findings show that *F. deltoidea* leaf varieties demonstrate potential as a good source of antioxidants. This study also showed that FDB is having a better potential to be further developed and used as nutraceutical agent comparative to other *F. deltoidea* varieties (FDM and FDS). The presence of coumaric acid and vitexin in the extracts may contribute to the antioxidative action of the plant, suggesting that the phenolic and flavonoid compounds present in the extracts could be responsible for its beneficial effects. Furthermore, the extracts are safe for consumption because they did not cause toxicity in the WRL68 normal liver cell line.

##  Supplemental Information

10.7717/peerj.5694/supp-1 Data S1 Raw data of antioxidant assays

10.7717/peerj.5694/supp-2 Data S2 Raw data of cytotoxicity assay

## Additional Information and Declarations

### Data Availability

The following information was supplied regarding data availability: The raw data are provided in a Supplemental File.
